# Disseminated Nocardiosis: Poorly Controlled Diabetes as an Immunocompromising State

**DOI:** 10.7759/cureus.84590

**Published:** 2025-05-21

**Authors:** Danielle G Iben, Emily Dobrzynski, Ritu Garg

**Affiliations:** 1 Internal Medicine, Loyola University Chicago Stritch School of Medicine, Maywood, USA; 2 Internal Medicine and Pediatrics, Loyola University Medical Center, Maywood, USA; 3 Internal Medicine, Loyola University Medical Center, Maywood, USA

**Keywords:** diabetes, disseminated nocardia, immunocompromised, infectious disease diagnosis, opportunistic bacterial infection

## Abstract

*Nocardia *species are environmental pathogens that cause rare infections, primarily affecting immunocompromised individuals. These infections can present as pulmonary, cutaneous, or disseminated diseases, often involving the central nervous system. The most common portal of entry is via inhalation or skin breakdown; it is often found in soil, water, and vegetable matter. While typically associated with organ transplantation, malignancy, and immunosuppressive therapy, this case highlights how poorly controlled diabetes may also function as an immunocompromised state, therefore increasing susceptibility to severe *Nocardia *infections.

We present a case of a 74-year-old man with poorly controlled type two diabetes mellitus (T2DM) with a hemoglobin A1c (HbA1c) greater than 8% who developed a diffuse vesicular rash, altered mental status, and generalized muscle aches. During hospitalization, imaging revealed bilateral lung nodules with cavitation and focal brain enhancement. He was initially treated for a disseminated varicella-zoster virus infection. However, further infectious workup, including molecular testing and tissue culture, confirmed *Nocardia* infection. The patient demonstrated significant clinical improvement with intravenous trimethoprim-sulfamethoxazole (TMP-SMX) and was discharged on a prolonged course of oral antibiotics.

This case focuses on uncontrolled diabetes as a potential immunocompromising condition that may increase the risk of *Nocardia* infection. The initial misdiagnosis underscores the diagnostic challenges of this rare disease, particularly in patients without overt immunosuppression. Given the rising global prevalence of diabetes, clinicians should maintain a broad differential when evaluating diabetic patients with systemic illness and unexplained neurological symptoms. Early recognition and targeted treatment of nocardiosis can prevent severe complications, including central nervous system involvement.

## Introduction

*Nocardia* species are environmental pathogens commonly found in soil, decaying organic matter, and water. Infections caused by these bacteria, collectively known as nocardiosis, are rare, with an estimated annual incidence of 500-1,000 in the United States [1]. Due to lack of surveillance, the true prevalence of this bacteria remains uncertain [1]. *Nocardia* are gram-positive, aerobic bacteria that may appear in tissue sections as branching, beaded rods [2]. Nocardiosis can manifest in pulmonary, cutaneous, or disseminated forms. Pulmonary involvement is the most common presentation, typically appearing as nodular or cavitary lesions on imaging, which can mimic tuberculosis or malignancy. Patients with nocardiosis may experience symptoms such as cough, fever, chest pain, and dyspnea. Disseminated disease frequently involves the central nervous system (CNS), most notably in the form of brain abscesses. These lesions often cause non-specific neurological symptoms, including headaches, altered mental status, seizures, or focal neurologic deficits. Cutaneous nocardiosis, though less common, typically follows traumatic inoculation and manifests as cellulitis, abscesses, or non-tender erythematous nodules, often with purulent drainage [1,3]. Although pulmonary and CNS involvement are the most common, nocardiosis can also lead to rare extrapulmonary complications, including cardiac involvement. Reported cases include pericarditis, pericardial effusion, and even constrictive pericarditis caused by *Nocardia* species, particularly in immunocompromised individuals. These less common disease manifestations carry a risk of significant morbidity if not recognized and treated promptly [1]. A definitive diagnosis of nocardiosis requires culture of the organism from a suspected site. *Nocardia *typically grows within two to seven days on most routine bacteriological media [4].

Nocardiosis classically is regarded as an opportunistic infection. Cell-mediated immunity is crucial for containing the infection, and a variety of immune-compromising conditions including organ transplant, cancer, diabetes mellitus, alcoholism, acquired immune deficiency syndrome, and long-term corticosteroid use have been reported to increase the risk of infection [5]. In studies involving nocardiosis, immunocompromised status was defined as having a history of hematologic or solid tumor malignancy, solid organ transplant, hematopoietic cell transplant, immunodeficiency, autoimmune/inflammatory disorder treated with immunosuppressive agents, recent chemotherapy, or high-dose corticosteroid use prior to diagnosis of nocardiosis [6,7]. Diabetes mellitus, particularly when poorly controlled, is known to impair immune function through mechanisms such as reduced neutrophil chemotaxis, phagocytosis, and microbial killing. Elevated glycated hemoglobin (HbA1c) levels have been associated with increased susceptibility to infection in large cohort studies [8-12]. However, despite this well-established link between hyperglycemia and immune dysfunction, diabetes is not commonly cited as a predisposing factor in case reports of nocardiosis. This may reflect under-recognition of its role or stricter historical definitions of immunocompromised status in the literature about *Nocardia*. Nonetheless, poorly controlled diabetes remains a plausible and clinically relevant factor in cases lacking traditional risk factors. Currently reports of immunocompromised patients have been the only population to develop cavitation and were more likely to have disseminated infections, particularly among organ transplant recipients. Despite similar clinical presentations between groups, immunocompromised patients had significantly higher mortality, demonstrating the greater severity and risk of nocardiosis in this population [6].

## Case presentation

A 74-year-old man with a history of poorly controlled type 2 diabetes mellitus (T2DM) with a HbA1c of 8% (8.4%-8.6% in last three years) while on medication, hypertension, and a well-differentiated rectal neuroendocrine tumor (NET) (status post-endoscopic mucosal resection in 2022, with no evidence of recurrence) presented in mid-November of 2024 with diffuse vesicular rashes, generalized muscle aches, and altered mental status. He lives in Durango, Mexico, and visits the United States twice a year to see his family. He works on a ranch spending more than 10 hours each day harvesting corn. The ranch has cows, though he does not spend much time with the animals. While there are others on the property who keep chickens, he generally does not work with them. He reported no known sick contacts and recalled a history of chickenpox during childhood. He had been working in the fields in Mexico for more than 10 hours daily prior to symptom onset.

**Figure 1:**
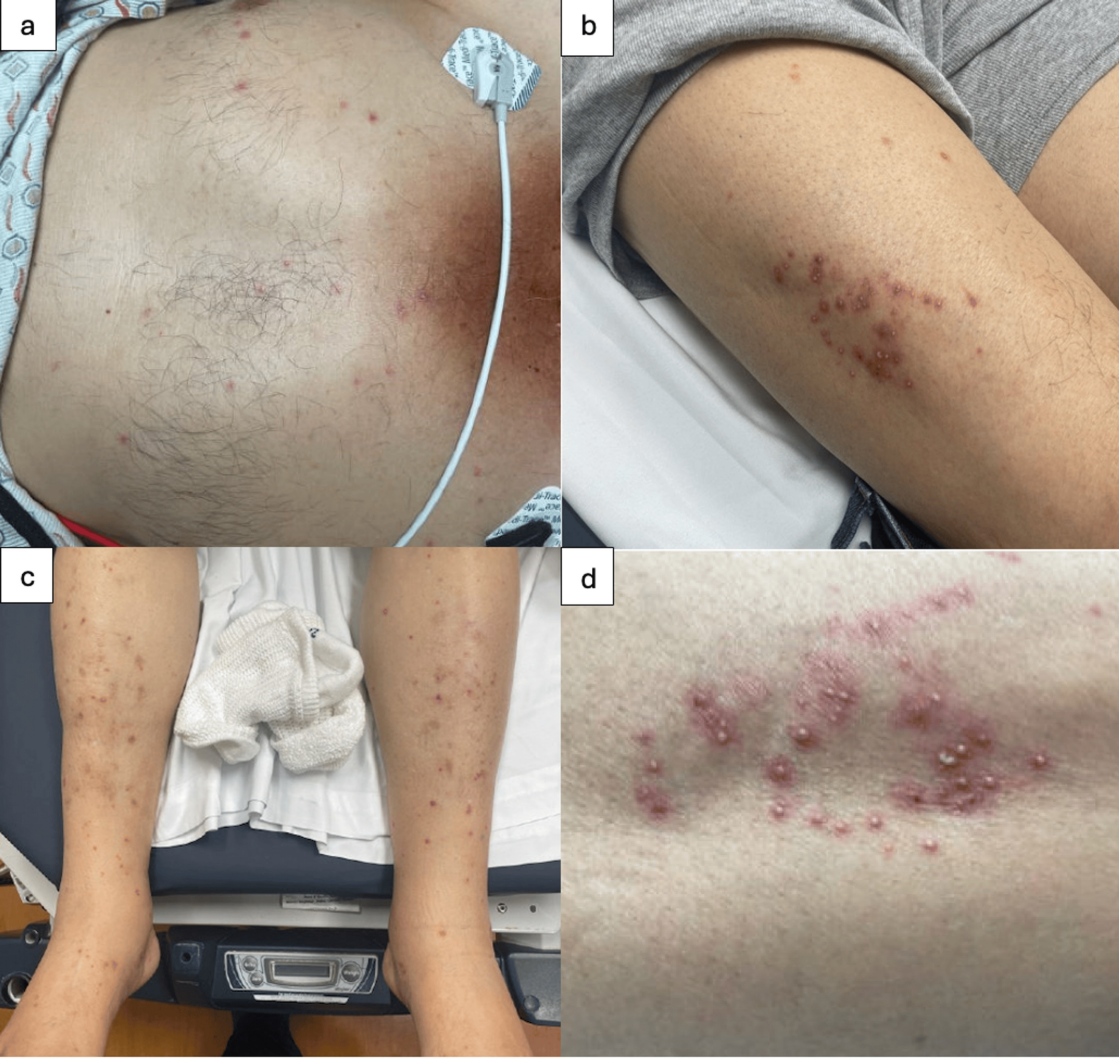
Diffuse vesicular rash involving multiple body sites. (a) Chest; (b) right upper thigh; (c) bilateral shins; (d) right upper thigh.

Approximately five days prior to admission, he developed diffuse muscle aches, which progressed to difficulty walking. Two days later, he developed a pruritic rash on his thigh that spread to most of his body. While still in Mexico, he was prescribed valacyclovir by a local physician due to concern for varicella-zoster virus (VZV) and had been taking it for two days before presenting to the hospital. His family had brought him straight from the airport to the hospital. On admission, his rash was non-pruritic and painless (Figure 1). He denied fevers, chills, gastrointestinal symptoms, or oral lesions. His home diabetes regimen included 22 units of glargine nightly, 25 mg of empagliflozin daily, 1000 mg of metformin twice daily, and 15 mg of pioglitazone daily, prescribed since 2021. However, adherence to this regimen was unclear. The oldest documented HbA1c was 7.6% in 2021, but since 2022, it had consistently been above 8%, indicating persistent uncontrolled diabetes.

On admission, his vital signs were: blood pressure of 173/80 mmHg, heart rate of 105 beats per minute, temperature of 98.4°F, and oxygen saturation of 98% on room air. Physical examination revealed a diffuse pustular rash with clusters on the anterior right thigh, bilateral pitting edema of the lower extremities, neck stiffness and tenderness in the thighs and forearms. He had significant dirt accumulation under his fingernails, suggesting regular contact with soil.

Laboratory testing was notable for leukocytosis with a markedly elevated absolute neutrophil count and a high neutrophil-to-lymphocyte ratio, suggesting a systemic inflammatory or stress response consistent with bacterial infection or other inflammatory condition. Respiratory viral panel, including influenza, respiratory syncytial virus (RSV), and COVID-19, was negative along with a test for methicillin-resistant *Staphylococcus aureus *(MRSA). Urinalysis showed no evidence of infection; however, ketones and glucose were detected. Additional findings included hyperglycemia, elevated inflammatory markers (C-reactive protein and lactate), elevated creatine kinase (CK), mild transaminitis, and mildly impaired renal function (Table 1).

**Table 1:** Initial Lab Values

Test	Value	Reference
White blood cell count	23.1 K/UL	3.5-10.5 K/UL
Granulocyte count	20.8 x 10⁹/L	1.5-7 x 10⁹/L
Granulocyte percentage	90%	40-60%
Eosinophil count	0 x 10⁹/L	0-0.7 x 10⁹/L
Neutrophil-to-lymphocyte ratio	29.71	0.78-3.53
Absolute neutrophil count	21.252 x 10⁹/L	<15 x 10⁹/L
Glucose	15.1 mmol/L	3.89-5.55 mmol/L
C-reactive protein	3,294.8 nmol/L	<77.2 nmol/L
Lactate whole blood	2.5 mm/L	0.9-1.7 mm/L
Creatine kinase	832 U/L	50-320 U/L
B-natriuretic peptide	237 ng/L	1-100 ng/L
High-sensitivity troponin I	25 ng/L	0-20 ng/L
Alkaline phosphatase	131 U/L	30-110 U/L
Alanine aminotransferase	45 U/L	10-40 U/L
Aspartate aminotransferase	61 U/L	15-45 U/L

Given the patient’s altered mental status and diffuse vesicular rash, there was a high clinical suspicion for disseminated VZV infection with possible CNS involvement. Although he reported a childhood history of chickenpox, the rash pattern and systemic symptoms raised concern for reactivation. A presumptive diagnosis of disseminated VZV encephalitis was made, and he was empirically started on intravenous (IV) acyclovir, piperacillin-tazobactam, and vancomycin. This was later narrowed to acyclovir, ampicillin, and ceftriaxone as per infectious disease physician recommendations. Lumbar puncture was deferred due to the diffuse rash over the lower back. A quantitative VZV polymerase chain reaction (PCR) was sent, and skin biopsies were obtained for further evaluation. Dermatology team was consulted and tissue cultures were sent.

**Figure 2:**
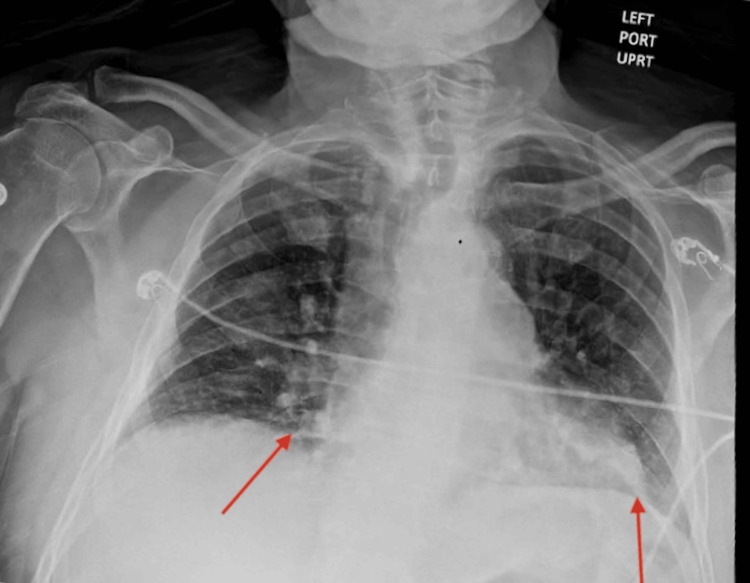
Anterior-posterior chest X-ray demonstrating hypoinflated lungs with bibasilar opacities (red arrows), likely representing atelectasis. Infectious processes could not be excluded.

The patient’s diffuse myalgias and significantly elevated CK raised concern for rhabdomyolysis or viral myositis. Differential considerations included exertional rhabdomyolysis, disseminated VZV, and dengue fever, given recent travel. Although the rash was not typical for dengue, its association with myalgias and viral myositis warranted evaluation. A broadened infectious workup was ordered including rapid plasma reagin (RPR), dengue virus PCR and serology, *Brucella* antibodies and culture, and *Salmonella* serology. HIV serology was sent due to concern for disseminated VZV. Early results for VZV, HIV, syphilis, and common respiratory pathogens were negative. Testing for *Brucella* and *Salmonella *- prompted by the patient’s frequent contact with soil, livestock, and unpasteurized dairy - resulted later and was negative. Initial imaging included a chest X-ray (Figure 2) showing hypoinflated lungs with bibasilar opacities, likely atelectasis, though infection could not be ruled out. A non-contrast head CT performed on admission to evaluate encephalopathy showed no acute intracranial pathology.

**Figure 3:**
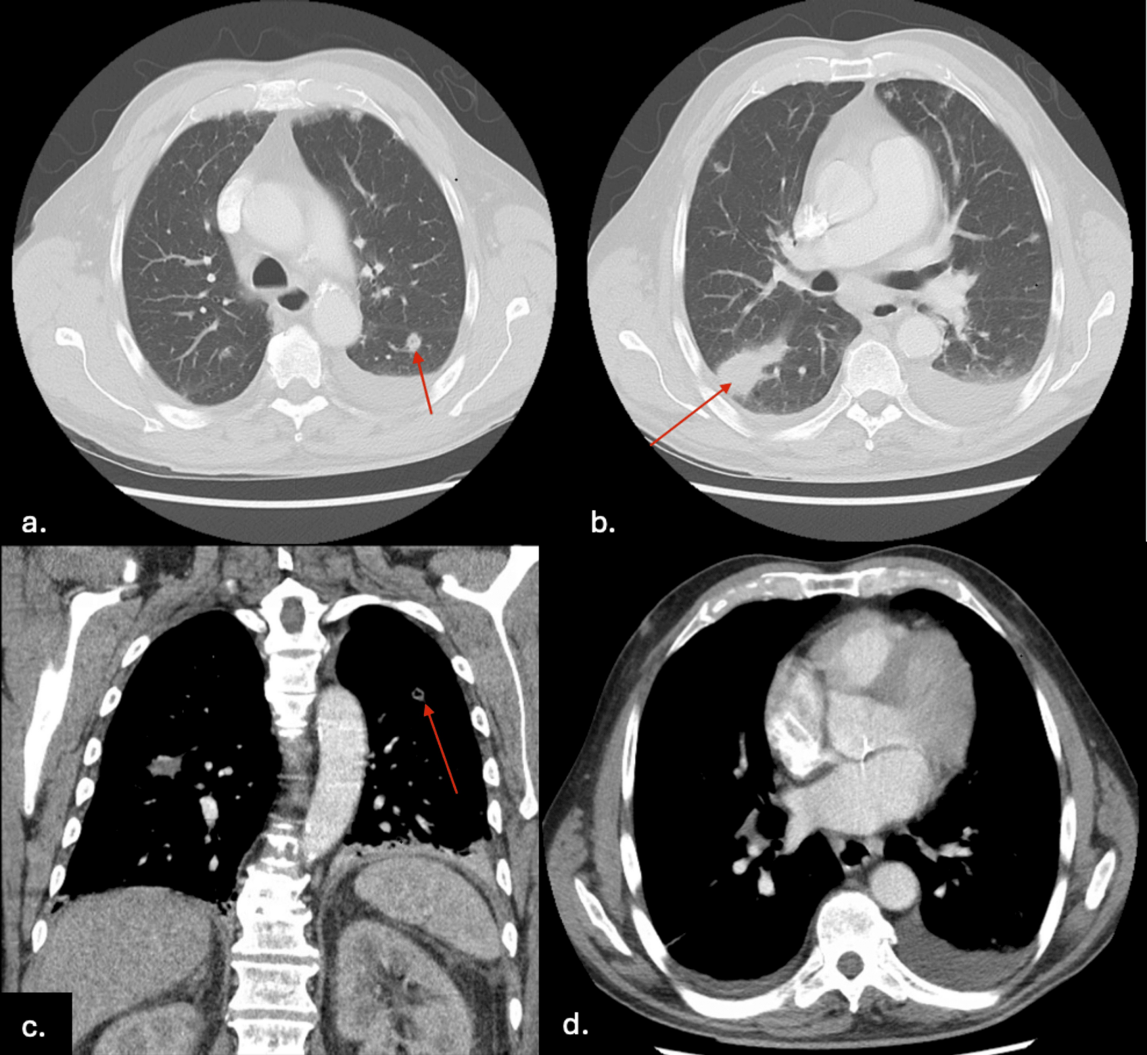
CT chest revealing diffuse bilateral nodular opacities, several with internal cavitation, suggestive of an infectious or inflammatory process. (a) Axial view: red arrow highlighting cavitation in the left lower lobe. (b) Axial view: dominant consolidation along the right oblique fissure and red arrow pointing to consolidation in the superior segment of the right upper lobe. (c) Coronal view: red arrow indicating cavitation in the left lung. (d) Axial view: Bilateral pleural effusions

After several days of hospitalization, the diffuse vesicular rash involving the face, extremities, and trunk began to crust and scab, with mixed stages of healing and no new lesions. Muscle strength was also beginning to improve. However, encephalopathy persisted, and the white blood cell count, although slightly improved, remained elevated. A chest CT performed one week after admission (Figure 3) demonstrated diffuse bilateral nodular opacities with subtle internal cavitation and a dominant consolidation along the right major fissure, findings suggestive of an infectious or inflammatory process. Around this time, VZV PCR returned negative. Blood cultures and all prior infectious workup had also remained negative, prompting further consideration of alternative diagnoses.

**Figure 4:**
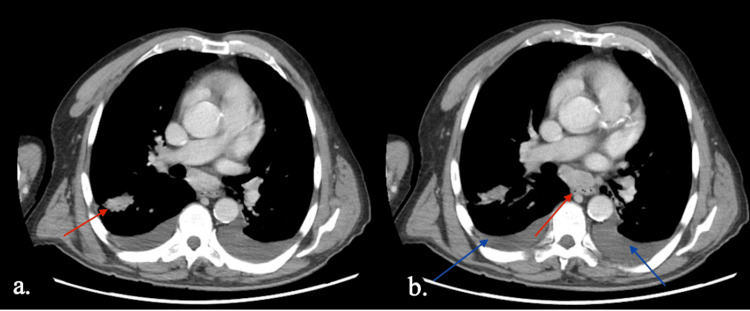
Follow-up CT chest consistent with known nocardiosis. (a) Scattered nodular and consolidative opacities in both lungs; red arrow indicates stable consolidation in the right oblique fissure. (b) Subcarinal presumed reactive lymphadenopathy (red arrow) and small pleural effusions, more prominent on the left (blue arrow).

**Figure 5:**
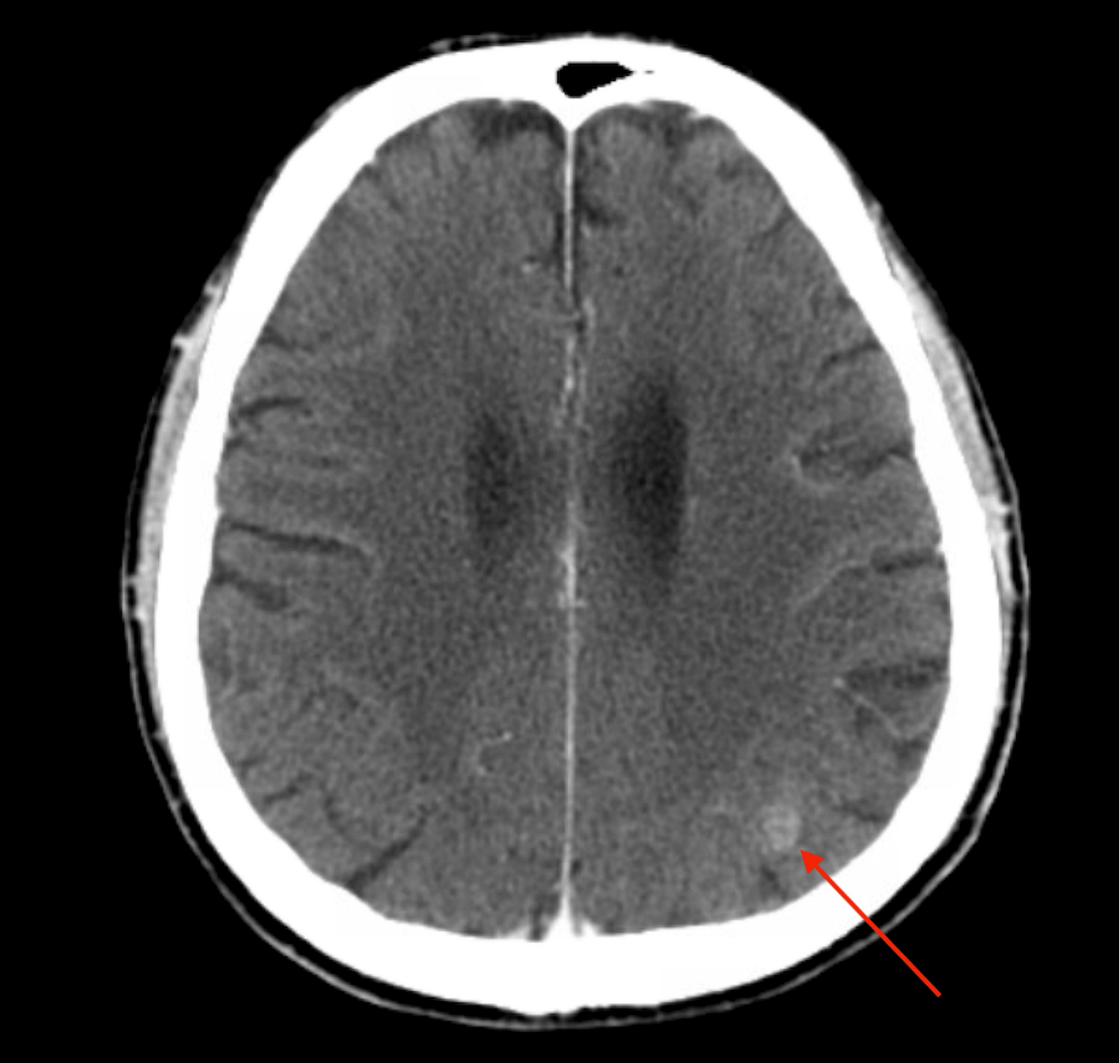
CT head showing contrast enhancement in the left parietal lobe (red arrow), suggestive of CNS involvement.

The following day, tissue culture from the cutaneous lesions grew *Nocardia brasiliensis and Nocardia vulneris*, and Karius molecular testing, sent concurrently, confirmed the diagnosis of disseminated nocardiosis. With this diagnosis established, repeat imaging was performed, including CT chest, abdomen, and pelvis (Figure 4) and CT head (Figure 5), which identified scattered lung nodules with cavitation, small pleural effusions, presumed reactive lymphadenopathy, and a focus of enhancement in the left parietal lobe suggestive of CNS involvement. Based on antibiotic sensitivities, his antimicrobial regimen was adjusted to IV trimethoprim-sulfamethoxazole (TMP-SMX) and ceftriaxone. The patient received 240 mg IV TMP-SMX every eight hours and 2 g ceftriaxone every 24 hours. He demonstrated significant clinical improvement following this change.

**Figure 6:**
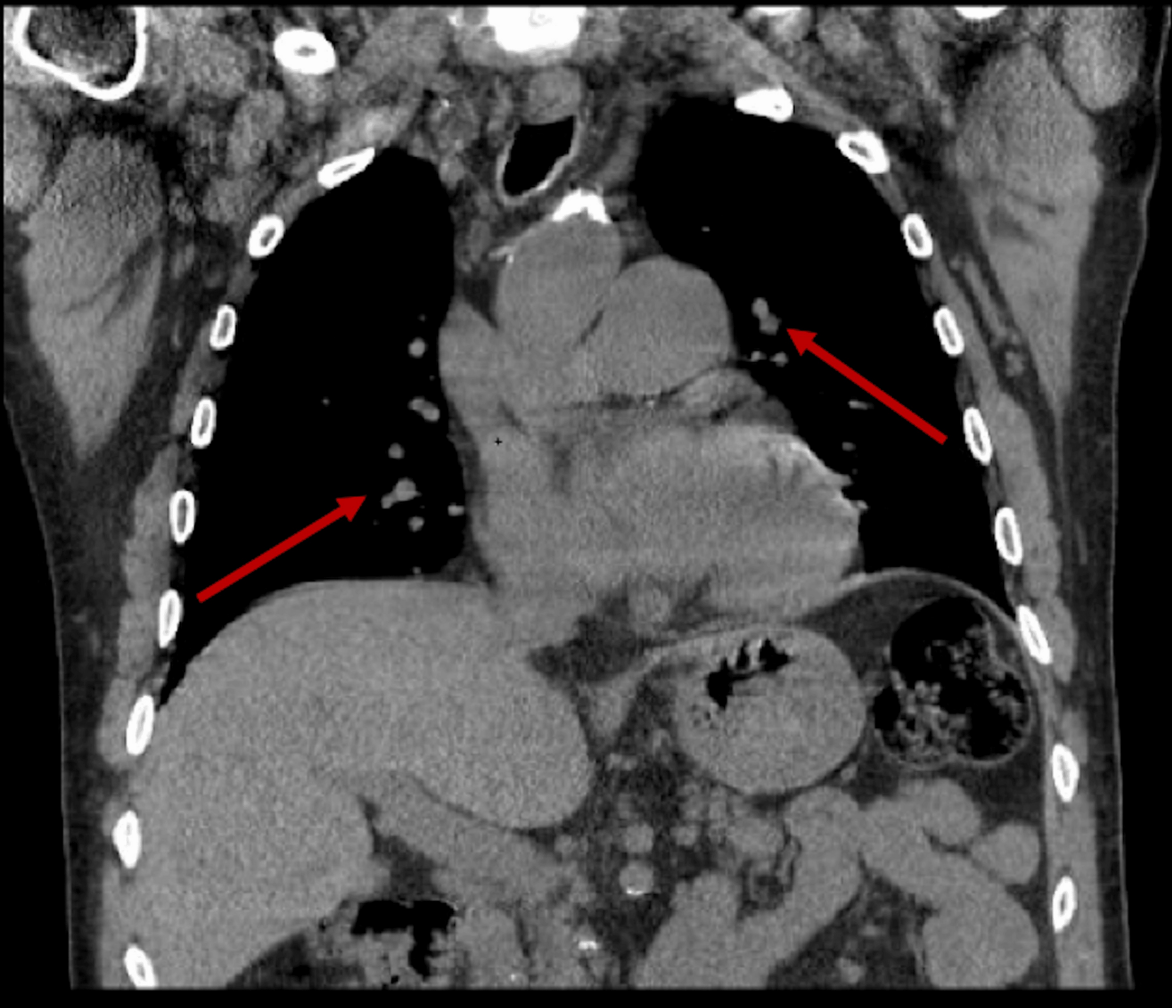
Follow-up CT chest demonstrating persistent but slightly improved bilateral reticulonodular opacities (red arrows).

**Figure 7:**
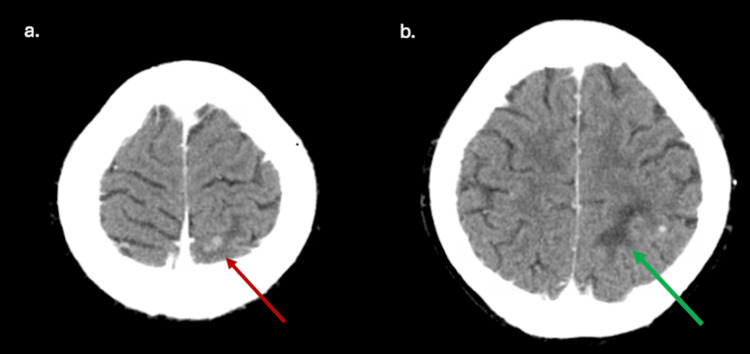
CT head demonstrating edema in the left parietal region with nodular foci of enhancement (red arrow) and possibly dilated adjacent vasculature (green arrow), concerning for evolving CNS infection.

**Figure 8:**
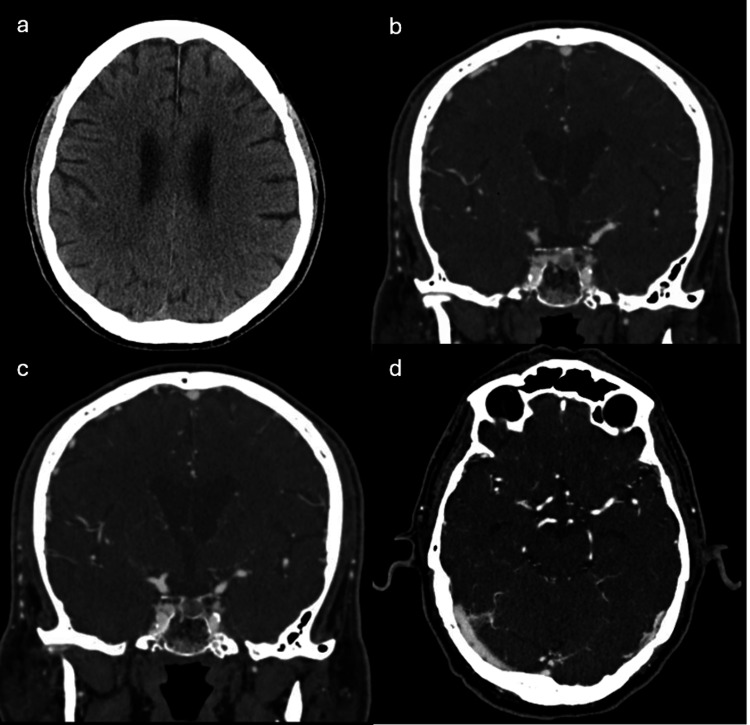
Non-contrast CT head showing interval resolution of previous left parietal edema and associated nodular focus of enhancement (a). CT Head Angiography reveals no evidence of mycotic aneurysm, high-grade stenosis or occlusion (b, c, d).

**Figure 9:**
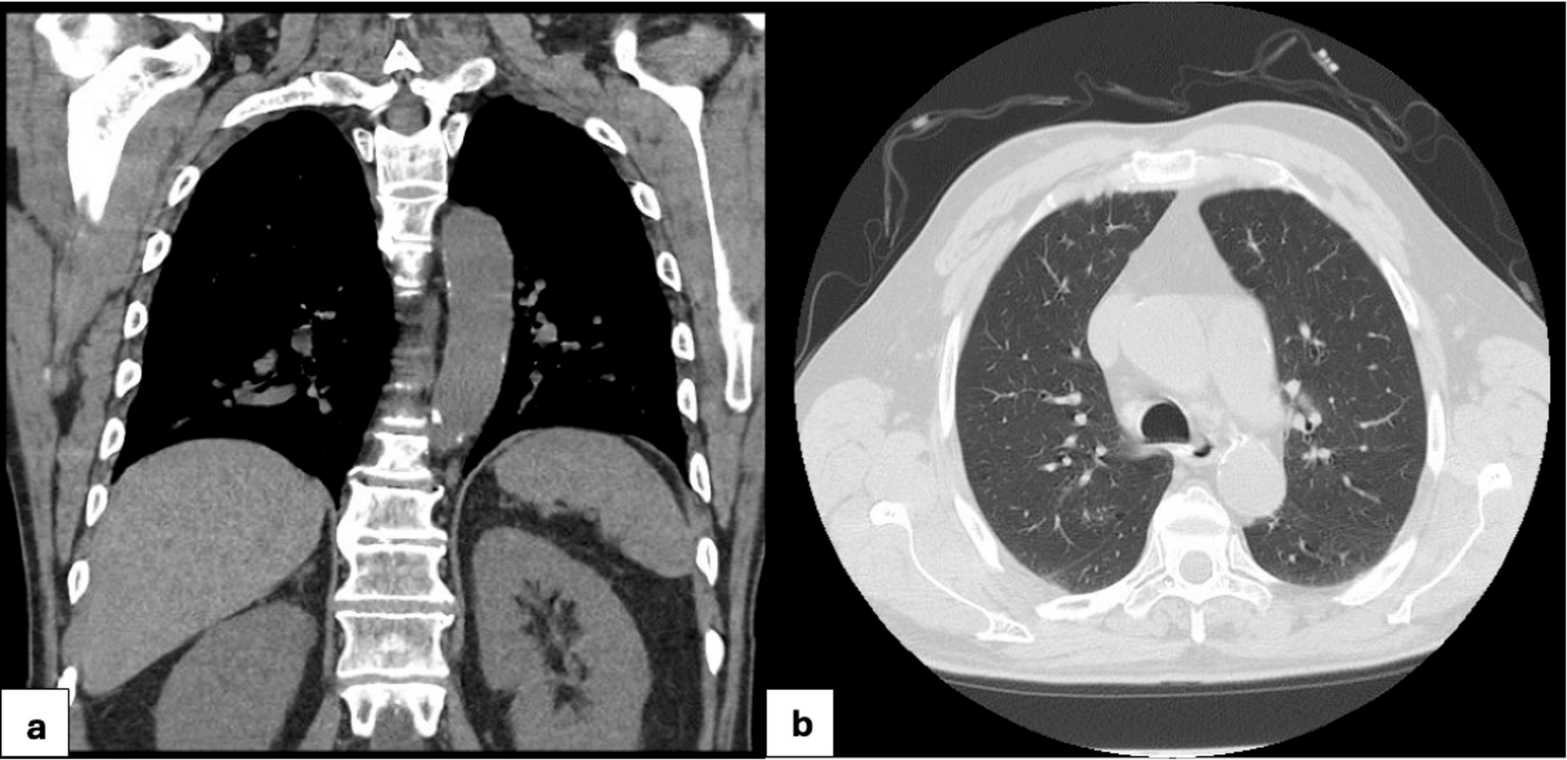
Final CT chest showing mild residual reticulonodular changes (b), likely post-inflammatory. Large airways remain clear (a).

He was discharged on 240 mg IV TMP-SMX every eight hours and 2 g ceftriaxone every 12 hours for six weeks, followed by oral TMP-SMX for at least six months. At outpatient follow-up six weeks after completing IV antibiotics, the patient demonstrated complete resolution of skin lesions and mentation. CT chest showed persistent but improved bilateral reticulonodular opacities (Figure 6). However, follow-up CT head (Figure 7) revealed residual edema in the left parietal region with nodular enhancement and possible dilation of nearby vessels, raising concern for a mycotic aneurysm. CT angiography was recommended. Repeat imaging performed approximately three months after initial head imaging demonstrated resolution of left parietal edema and the associated nodular focus on CT head, with no evidence of mycotic aneurysm on CT angiography (Figure 8). A final chest CT showed resolution of prior cavitary nodules and clear airways (Figure 9).

## Discussion

Diagnostic reasoning and initial workup

The diagnostic challenges in this case underscore the importance of maintaining a broad differential in patients presenting with ambiguous symptoms suggestive of opportunistic infection. Disseminated VZV was initially considered given the combination of diffuse vesicular rash, encephalopathy, and elevated CK, which raised concern for viral myositis or rhabdomyolysis. Dengue fever was also entertained due to the patient’s recent travel history and diffuse myalgias, although the rash was not consistent with classical dengue [13,14]. Initial testing focused on more common infectious causes, including urinary tract infections, influenza, RSV, COVID-19, and MRSA, all of which were negative. Based on the broader clinical picture and risk factors, an expanded infectious workup was initiated including testing for VZV, HIV, syphilis, dengue virus, *Brucella*, and *Salmonella*. HIV testing was obtained given the concern for disseminated VZV and the overall clinical presentation. While HIV itself does not typically cause a vesicular rash, it can predispose patients to reactivation or dissemination of vesicular viral infections such as VZV. As such, evaluation for underlying immunosuppression was clinically warranted. Given the patient’s geographic and occupational exposures - including regular contact with soil, livestock, and consumption of unpasteurized dairy products - the infectious workup was broadened to include *Salmonella* and *Brucella* serologies, which ultimately returned negative after the diagnosis of nocardiosis had already been confirmed via tissue culture [14].

This stepwise diagnostic approach was guided by the patient’s evolving clinical picture - including rash, altered mental status, and elevated CK - which raised early concern for disseminated viral or atypical infection and justified targeted imaging and expanded infectious testing.

Confirmation and diagnostic yield of imaging

The diagnosis of nocardiosis was confirmed through tissue culture, prompting further imaging that demonstrated multisystem involvement, including cavitary lung nodules and a CNS lesion. Given the rarity of nocardiosis, it would be neither practical nor cost-effective to pursue exhaustive testing and imaging in every patient with uncontrolled diabetes [3]. Instead, a detailed history, particularly focusing on environmental exposures, occupational risks, and immunocompromising status, paired with clinical clues such as non-resolving pneumonia, cavitary lesions, or unexplained neurologic symptoms, should guide further investigation. *Nocardia* species are widely distributed in nature, found in diverse ecological settings including soil, water, and decaying vegetation, with increased prevalence in tropical and subtropical climates [1]. Although nocardiosis is classically considered an opportunistic infection occurring in immunocompromised hosts - including those with hematologic malignancies, HIV, solid organ or stem cell transplants, or chronic corticosteroid use - it can also occur in immunocompetent individuals, especially in the setting of relevant environmental exposures [1,3]. Imaging plays a critical role not only in detecting characteristic lesions, such as pulmonary or cerebral cavities, but also in evaluating the extent of dissemination, which informs both prognosis and treatment duration. For example, pulmonary nocardiosis may require six months of antibiotics, while CNS involvement may necessitate up to 12 months [1,4]. While comprehensive imaging should not be performed indiscriminately, it is a valuable tool when clinical suspicion is high - particularly in the presence of systemic symptoms without clear etiology. A practical and cost-conscious approach involves correlating clinical signs and symptoms with risk factors - such as environmental exposure or immunocompromised state - and then selectively employing testing and imaging to confirm diagnosis and assess disease burden.

Microbiological and molecular testing

Definitive diagnosis of *Nocardia* infection is made by gram stain or culture from sputum or tissue biopsies. However, as a slow-growing organism, cultures often require prolonged incubation times [1,15]. Molecular testing can enhance diagnostic accuracy and allow for earlier initiation of treatment. A multicenter study found that PCR testing of tissue samples, most commonly bronchoalveolar lavage, had a sensitivity of 88% and specificity of 74% for diagnosing nocardiosis in immunocompromised patients [4].

Metagenomic next-generation sequencing tools, such as the Karius test, are increasingly used to detect opportunistic pathogens due to their broad organism coverage, rapid turnaround, and noninvasive sampling [4]. In our case, the Karius test identified *Nocardia* days before culture confirmation, allowing for timely initiation of therapy. Nevertheless, culture remains the gold standard, particularly for enabling antimicrobial susceptibility testing and guiding long-term management [4].

Diabetes as an immunocompromising condition

This case further illustrates the potential for uncontrolled diabetes to serve as an immunocompromising condition, increasing susceptibility to opportunistic infections such as disseminated nocardiosis. Although nocardiosis is classically associated with profound immunosuppression such as in transplant recipients or individuals receiving chemotherapy, our patient lacked these traditional risk factors [5,6,7]. Poorly controlled diabetes (HbA1c of 8%) was the only clinical variable that may have increased susceptibility.

The patient’s history of a well-differentiated rectal NET is clinically relevant, as malignancy can confer some degree of immunocompromise. However, the risk is typically linked to active disease or immunosuppressive treatment. In one case series, all nine patients with malignancy-associated nocardiosis were either undergoing chemotherapy or receiving corticosteroids at diagnosis - none were treatment-naïve [16]. Similarly, a surveillance study found that while 22% of nocardiosis cases had a malignancy history, most involved hematologic cancers or patients who had received stem cell transplants or monoclonal antibody therapies such as alemtuzumab or rituximab, which impair cell-mediated immunity [1]. Our patient’s tumor-a low-grade, 0.7-cm well-differentiated NET confined to the submucosa (pT1a), with a mitotic index of <1 per 2 mm², a Ki-67 proliferation index <1%, and no lymphovascular or perineural invasion-was completely resected via endoscopic mucosal resection with negative margins. Immunohistochemistry confirmed neuroendocrine differentiation (positive for synaptophysin and CD56, negative for chromogranin A), and follow-up evaluation showed no evidence of recurrence. Evidence suggests that tumor-induced immunosuppression can reverse after resection, even in cases of metastatic disease [17], with more recent data indicating that systemic immune function may normalize within weeks of tumor removal [18].

While a prior malignancy should always prompt careful history-taking and consideration in the diagnostic process, the absence of active disease and immunosuppressive therapy in this case makes it an unlikely contributor to ongoing immune dysfunction. Therefore, although cancer history remains an important part of the clinical context, poorly controlled diabetes stands out as the most plausible and clinically relevant factor predisposing this patient to opportunistic infection.

Although an HbA1c of 8% is considered moderately uncontrolled, several large-scale studies have demonstrated that even this level of hyperglycemia can meaningfully increase susceptibility to infection. A retrospective cohort study of over 85,000 adults with type 2 diabetes found that the risk of infection-related hospitalization rose significantly when HbA1c exceeded 7%, with the greatest risk observed in patients with values ≥11% [8]. Similarly, recent cohort data from over 26,000 patients with type 1 diabetes showed that increasing HbA1c levels were strongly correlated with infection risk, even after adjusting for age, sex, and ethnicity [9].

Mechanisms of immune dysfunction

These epidemiological findings align with the growing recognition that diabetes, though not traditionally classified as a cause of severe immunocompromise, significantly impairs host immunity. Hyperglycemia disrupts neutrophil chemotaxis, phagocytosis, and reactive oxygen species production, weakening the innate immune response and increasing susceptibility to intracellular pathogens like *Nocardia* [10]. Chronic low-grade inflammation and T-cell dysfunction further impair immune surveillance, allowing pathogens to evade clearance and establish infection [11]. These mechanisms raise important questions about whether patients with poorly controlled diabetes should be viewed as immunocompromised in the context of opportunistic infections.

Clinical implications and future considerations

*Nocardia's* ability to survive intracellularly and disseminate, most commonly to the lungs and CNS, amplifies the risk in patients with impaired immune defenses [1]. Beyond *Nocardia*, uncontrolled diabetes has also been associated with other rare opportunistic pathogens, including *Rhizopus* in mucormycosis and *Burkholderia pseudomallei* in melioidosis [19,20].

Given the growing prevalence of diabetes worldwide, projected to affect over 500 million people by 2035, the potential for increased susceptibility to *Nocardia* and other opportunistic infections warrants clinical attention [11]. A retrospective study examining 44 patients with nocardiosis who were considered immunocompetent suggests that diabetes may have been an underrecognized risk factor in some cases [12]. This invites discussion about whether clinicians should adopt a higher index of suspicion for atypical infections in patients with diabetes, particularly when evaluating cases of unexplained systemic illness.

Despite well-established guidelines for managing microvascular and macrovascular complications of diabetes, its role in infection risk remains underemphasized [10,12]. A shift in perspective may influence how we approach diagnostic workups in diabetic patients with ambiguous presentations, as opportunistic infections may be more common in this population than currently appreciated. Given the ubiquity of diabetes and its broad effects on immune function, it is worth considering whether we should routinely expand the infectious disease differential in diabetic patients presenting with unexplained systemic symptoms. Further research is needed to determine whether modifying how we evaluate these patients could lead to earlier diagnoses, more targeted treatments, and ultimately better clinical outcomes.

## Conclusions

This case illustrates the importance of maintaining a broad differential diagnosis in diabetic patients with unexplained systemic illness, particularly when other risk factors, such as environmental exposures or a history of malignancy, are present. While disseminated nocardiosis remains rare, its potential severity highlights the need for thorough history-taking and attention to subtle clinical clues, rather than indiscriminate testing. In our patient, the absence of active malignancy or immunosuppressive therapy made poorly controlled diabetes the most plausible contributor to immune dysfunction.

Rather than advocating for comprehensive imaging or microbiologic testing in all patients with diabetes, we emphasize a targeted, symptom-driven approach. Molecular tools like PCR and metagenomic sequencing can complement traditional cultures and may expedite diagnosis when the suspicion of opportunistic infection is high. Imaging, while not always warranted initially, plays a critical role in assessing the extent of dissemination and guiding treatment duration once a diagnosis is suspected.

As diabetes becomes increasingly prevalent, clinicians should remain vigilant for atypical infections in this population and consider opportunistic pathogens when standard treatments fail or symptoms remain unexplained. A more nuanced understanding of uncontrolled diabetes as a potential immunocompromising condition may lead to earlier diagnoses, more precise treatment strategies, and ultimately improved outcomes.
